# Mental and Physical Health Correlates of Financial Difficulties Among African-American Older Adults in Low-Income Areas of Los Angeles

**DOI:** 10.3389/fpubh.2020.00021

**Published:** 2020-02-12

**Authors:** Meghan C. Evans, Mohsen Bazargan, Sharon Cobb, Shervin Assari

**Affiliations:** ^1^Department of Family Medicine, Charles R. Drew University of Medicine and Science, Los Angeles, CA, United States; ^2^Department of Family Medicine, University of California Los Angeles (UCLA), Los Angeles, CA, United States; ^3^School of Nursing, Charles R. Drew University of Medicine and Science, Los Angeles, CA, United States

**Keywords:** race/ethnicity, African Americans, ethnic groups, depressive symptoms, self-rated health, chronic medical conditions, financial difficulties, older adults

## Abstract

**Background:** While financial difficulties correlate with mental and physical health status, less is known about these associations among economically disadvantaged African-American (AA) older adults.

**Objective:** This study explored mental and physical health correlates of financial difficulties among AA older adults in low-income areas of south Los Angeles.

**Methods:** A cross-sectional study on 740 AA older adults (age ≥ 55 years) conducted in South Los Angeles between 2015 and 2018. Independent variable was financial difficulties. Outcomes were depressive symptoms, chronic pain, chronic medical conditions, self-reported health, and sick days. Age, gender, educational attainment, living alone, marital status, smoking, and drinking were also measured. Zero order (unadjusted) and partial (adjusted) correlates of financial difficulties were calculated for data analysis. Adjusted (partial) bivariate correlations controlled for age, gender, education, marital status, living alone, and health insurance.

**Results:** In adjusted analyses, financial difficulties were positively associated with chronic pain, chronic medical conditions, self-rated health, sick days, and depressive symptoms.

**Conclusion:** Financial difficulties seem to be linked to chronic pain, chronic medical conditions, self-rated health, sick days, and depressive symptoms. The results advocate for evaluation of social determinants of health in providing health care of AA older adults. Addressing financial difficulties may help with the health promotion of low-income AA older adults in urban areas.

## Introduction

Socioeconomic as well as racial/ethnic disparities are widely acknowledged in the United States. The median household income of Whites in the United States is $65,041 compared to the median income of African American (AA) households at $39,490 (1). Furthermore, 22% of the AA population live below the poverty line compared to 11% of Whites. (1). Additionally, AA populations suffer disproportionately from health issues such as chronic medical conditions (CMCs) and chronic pain (2–5). While research has measured the relationship between financial difficulties and a variety of individual health outcomes (6–8), there is still much to be understood about the role of financial difficulties in the mental and physical health of older adults and racial/ethnic minority populations. Considering the economic and health disparities facing AA populations, it is crucial to understand how a variety of health outcomes relate to experiences of financial difficulties in AA populations. The current study seeks to add to the literature by exploring multiple mental and physical health correlates of financial difficulties in older AA adults, while controlling for relevant demographic and social factors.

### Mental and Physical Correlates of Financial Difficulties

For certain health behaviors, such as drinking alcohol, financial difficulties appear to be associated with increased risk. A meta-analysis and systematic review of the literature reveals that debt is related to an increased odds of problem drinking (8). Additionally, research indicates chronic financial difficulties have a direct positive effect on drinking as a coping mechanism, which in turn has a direct effect on alcohol consumption and alcohol-related problems, such as binge drinking (8, 9). Furthermore, this research found that the relationship between drinking to cope was more strongly associated with alcohol consumption and related problems for AAs compared to non-AAs (9). Research among the elderly has found that although greater health problems are related to abstinence from alcohol, financial difficulties have the opposite effect, increasing odds of alcohol use among older adults (10).

A systematic review of the literature by Richardson and colleagues revealed a number of findings indicating a relationship between financial concerns and the likelihood to smoke (8). Recent findings indicate financial difficulties increase the risk of tobacco/nicotine use, and this relationship is especially strong for AA populations (11). Furthermore, financial difficulties can influence smoking cessation such that pre-quit financial difficulties are associated with more severe withdrawal symptoms which in turn reduces the likelihood of smoking abstinence (12, 13). Overall, the research indicates smoking is likely associated with financial difficulties in a way that may be especially strong for AA samples and therefore this health behavior is included in our current model of determinants.

Depressive symptoms have also been associated with financial difficulties, which can vary by race/ethnicity (14). A meta-analysis of the literature revealed more severe debt is associated with increased odds of depression (8). Furthermore, research specifically on AA populations has also found financial difficulties are associated with greater depressive symptoms (15, 16). Financial difficulties are more strongly related to major depressive episodes for AA adults rather than Whites (14). Furthermore, there may be an increased risk for older adults, considering research demonstrating disparities in depression across SES levels seems to widen across the lifespan, indicating an increased risk of low-income older adults to develop symptoms (17). Therefore, it is imperative that we include depressive symptoms as a correlate of financial difficulties in our sample of older AA adults.

Physical health may be measured through a variety of variables including chronic pain, CMCs, self-rated health (SRH), and sick days. Each of these health factors may be important correlates of financial difficulties within an older AA adult sample.

Chronic pain has been associated with financial difficulties in a variety of studies. Greater disability and distress from chronic pain is associated with living in low socioeconomic areas (18). Research has also demonstrated that greater severity and disability due to pain (19, 20) as well as greater risk of chronic pain following injuries (21) are related to low income levels. The incidence of chronic pain is also associated with financial difficulties (22). Demonstrating the relationship between SES and chronic pain, Dorner et al. found evidence that individuals in low SES groups, determined by measures including income, felt two to three times more disabled by the same level of severity of pain than those in the highest SES group (19).

In addition to chronic pain, it appears financial difficulties and CMCs may be related as well. In AA samples, financial difficulties have been linked to the increased risk of diagnosis of certain CMCs, such as Type 2 diabetes (23). Additionally, a study of older women found that those reporting financial difficulties were about 60% more likely to die within the next 5 years (24). Not only do financial difficulties predict morbidity and mortality, but CMCs can in turn also increase risk of financial difficulties, as research on those affected by cancer showed that about a third of participants lost all or most of their savings (25).

In terms of SRH, a study of older adults showed poorer SRH was related to living in a neighborhood with a higher proportion of people below the poverty line, regardless of race/ethnicity (26). Studies of AA samples and low-income samples have also found financial difficulties are associated with poorer SRH (27, 28). Furthermore, in older adults, poor SRH can increase the likelihood of future financial strain (29).

Research has also indicated a relationship between financial difficulties and work absenteeism (30). Considerable research has indicated sick days as related to stressors, such as experiencing financial difficulties for both women (31) and men (32). Research has suggested that financial strain may mediate the link between depression and sick days (33). Based on this literature, chronic pain, CMCs, SRH, and sick days may all be important health indicators related to financial difficulties. The current study explores the relationship between financial difficulties and a variety of demographic, social, behavioral, and health factors, as well as specifically examines the mental and physical health correlates of financial difficulties in older AA adults living in low-income areas of south Los Angeles.

### Demographic, Social, and Behavioral Factors

Gender, like race/ethnicity, is another factor related to gaps in financial difficulties. In 2016, the female-to-male earnings ratio in the United States was 80.5%, and the median income for men was $51,640 compared to women at $41,554 (1). Furthermore, gender and financial difficulties may have a stronger relationship among AA populations. Recent data from the U.S. Census Bureau indicates 55% of AA children are in single-parent households, with the vast majority being led by single mothers (34). Data on AA single-mother households shows 43% were below the poverty line in 2012, compared to 22% of AA single-father households and 9% of married family households of any race/ethnicity (34). In recent decades, older African Americans, especially women, have emerged as custodial parents to their grandchildren, leading to worsening of health, social, and financial status and threatening their well-being (35, 36). Adding parental responsibilities in the later age can lead to added costs and burdens, especially if the child has chronic health issues (37) or if there is only one caregiving grandparent (38). Thus, in this study it is imperative to control for this factor when examining the relationship between mental and physical health factors and financial difficulties.

Age may also be related to financial difficulties in a variety of ways. Research indicates that especially for older AA women, financial difficulties predict mortality (24). Age can increase risk of health issues, such as cancer, which can also lead to increased financial difficulties (25). Additionally financial difficulties, such as health expenditures, can accumulate over the life-course, taking a toll on those at older ages (7). Furthermore, financial exploitation of older adults can be a common problem and being AA or residing in high-poverty areas places certain older adults at increased risk (6). Overall, age and gender appear to play a role in the risk for and consequences of financial difficulties, especially for AA populations, and therefore it is imperative to include these factors in our model and control for this demographic factor in our adjusted analysis.

Similar to the disparities in other SES indicators, educational attainment varies by race/ethnicity, which may be largely impacted by financial difficulties. While family assets positively impact the chances of college graduation for AA and White students, family debt is more detrimental to AA students likelihood of graduating than White students (39). Research from a national sample of low- and middle-income households found that, for AA students, the odds of student loan indebtedness is twice as high as it is for White students, and this disparity persists even after accounting for degree completion and other SES factors (40). The financial burdens of education are greater for AA students and their college attendance is significantly lower (41). Even though education is typically associated with increases in SES, recent research found that educational attainment is associated with increases in future income and emotional well-being for Whites but not AAs (42). Therefore, there is more to be parsed concerning the role of educational attainment in reducing financial difficulties in AA samples.

Data from the U.S. Census Bureau indicates an upward trend in living alone, especially among older adults, with 36.1% of women and 18.9% of men living alone over the age of 65 (34). While there appears to be much research indicating the link between living alone and psychological distress (43–45), our review of the literature indicates a gap in research on living alone and financial difficulties in the U.S., especially for older adults or racial/ethnic minorities (46, 47). Those who live alone may have sufficient income to support themselves, negating the need to live with others, or conversely those who live with others may be able to afford a higher standard of living than those living alone through combined income and resources. Given conflicting past research and current data, the current study aims to control for this potential confounding variable.

Marital status, on the other hand, has more often been studied in relation to financial difficulties. Living with a spouse is associated with decreased risk of financial exploitation for older adults (6). Even though research from a nationally representative sample indicates married people are less likely to declare bankruptcy than divorced or separated people, never-married people were the least likely (48). While marital status may be protective against some forms of financial stress, financial difficulties can also take a toll on relationship quality and increase risk of divorce (49). Among AA samples, couples who experience financial difficulties are less likely to act supportively toward their partners (50) and are at higher risk for decreased effective communication, relationship satisfaction, and relationship confidence (51). Considering the bidirectional relationship between marriage and financial difficulties, marital status should be included and controlled for in any study on mental and physical health correlates of financial difficulties.

### Aim

This study explored the relationship between financial difficulties and mental and physical health factors for this high-risk sample. The above literature review on mental and physical health as well as demographic, social, and behavioral correlates of financial difficulties was mainly derived from research performed in general population. We sought to extend this literature by exploring correlates of financial difficulties in economically disadvantaged older AA adults who have heightened risk for financial difficulties and health issues. In our analysis, we controlled for demographic and social factors.

## Methods

### Institutional Review Board (IRB)

Charles R. Drew University of Medicine and Science (CDU) Institutional Review Board (IRB) approved the study protocol. All participants signed a written informed consent prior to their enrollment and received financial incentive.

### Data Source

Between 2015 and 2018, a cross-sectional survey was conducted among older AA adults from low-income areas in south Los Angeles. We used convenience sampling from economically disadvantaged areas of south Los Angeles to recruit older AA adults from 16 predominantly AA churches, 11 senior residential units, and low-income public housing projects. Church leaders and apartment managers facilitated recruitment by encouraging eligible adults in their community to participate in this comprehensive health evaluation which would include collecting their demographic, social, behavioral, and health information. As part of the primary study, a comprehensive evaluation of each participant's medication was performed but this was not included in the current study.

Eligibility was restricted to those adults who were AA, 55 years or older, able to complete an interview in English, and resided in Service Planning Area 6 (SPA 6), one of the most economically disadvantaged urban areas in Los Angeles county as well as the highest percentage of AAs (27.4%) of the SPAs in Los Angeles (52). SPA6 has the highest unemployment rate (13.65%) as well as the highest percentage of household incomes <100% of the Federal Poverty Level (33.6%) of the SPAs in Los Angeles (52, 53). Those adults who were institutionalized, enrolled in other clinical trials, or had poor cognitive performance were excluded. The sample was 740 AA adults aged 55 or older.

### Measures

The current study collected data through structured face-to-face interviews using several pages of checklist-style questionnaires created by the investigator based on standard validated measures and previous research. Interviews took place over several hours and collected information concerning gender, age, educational attainment, living alone, marital status, health behaviors (smoking and drinking), depressive symptoms, chronic pain, CMCs, SRH, sick days, and financial difficulties.

#### Dependent Variable

##### Demographic factors

Gender was treated as a dichotomous variable (1 female, 0 male), and age was operationalized as an interval variable.

##### Educational attainment

Educational attainment was treated as an interval variable (years of schooling), with more years reflecting higher educational attainment.

##### Living arrangement and marital status

Living arrangements and marital status were treated as dichotomous variables (1 living alone, 0 living with someone else; 1 married, 0 not-married).

##### Smoking

Participants reported their current smoking status, by answering the question “How would you describe your cigarette smoking habits?.” Responses included current smoker, previously smoked, or never smoke. This variable was treated as a dichotomous variable (1 ever smoker, 0 never smoker).

##### Binge drinking

Participants reported their drinking status by answering questions such as “Do you drink alcohol?,” “Do you ever drink five or more drinks on one occasion?,” “In the past 30 days, how many times did you have five or more drinks on one occasion?” Responses to the first and second question were yes or no. Using these items, we defined a dichotomous variable that indicated any binge drinking.

##### Depressive symptoms

Depressive symptoms were measured using the 15 item- Short Geriatric Depression Scale (GDS) (54, 55). Responses were on a “yes” or “no” scale. Summary scores were calculated, ranging between 0 and 15, with higher scores reflecting more depressive symptoms. The GDS-Short form has been used extensively among older adults in community and clinical settings to measure depression and has shown excellent reliability and validity (54, 55).

##### Chronic pain

Chronic pain was measured using four subscales of the McGill Pain Questionnaire–Short Form 2 (MPQ-SF-2) (56–59). Over 22 items, participants rated the extent of their past-week experience of various types of pain on an 11-point numeric scale (0 none to 10 worst possible). The subscales of the MPQ-SF-2 include: (a) continuity (throbbing, cramping, gnawing, aching, heavy, and tender pain), (b) intermittence (shooting, stabbing, sharp, splitting, electric-shock, and piercing pain), (c) neuropathic nature (hot-burning, cold-freezing, itching, tingling or “pins and needles,” light touch, and numbness pain), and (d) affective domain (tiring-exhausting, sickening, fearful, and punishing-cruel pain). Based on the average responses to all questions, a total pain score was calculated (56–59), and a higher score reflected more chronic pain.

##### Number of CMCs

Participants reported whether a physician has ever told them that they have any of the following CMCs: hypertension, heart disease, diabetes, lipid disorder/hypercholesterolemia, cancer, asthma, osteoarthritis, thyroid disorder, chronic obstructive pulmonary disease, rheumatoid arthritis, and gastrointestinal disease. Although self-reports provide valid information regarding CMCs, some level of measurement bias is expected (60).

##### Self-rated health (SRH)

This study measured SRH by the question, “Would you say your health in general is excellent, very good, good, fair, or poor?” Responses included excellent (1), very good (2), good (3), fair (4), and poor (5). SRH was treated as a continuous variable with higher scores indicating worse health. Poor SRH has been demonstrated to be a strong predictor of mortality across settings (61). The Institute of Medicine suggests that SRH be used as a tool for monitoring the health of people in the United States in national studies (62).

##### Sick days

Sick days were measured through a single item which asks, “In the past 12 months, how frequently have you been sick?” Responses included never (1), rarely (2), sometimes (3), most of the time (4), and always (5). Sick days were treated as an interval variable, with a higher score indicating more sick days. Frequency of sick days is commonly used as a proxy for health status (63, 64).

##### Health insurance

Health insurance was also measured through a single item which asks, “Do you have health insurance?” and the type of insurance was also noted. Responses were treated as a dichotomous variable (yes or no). This variable was not included in the analysis, however, as 98.8% of participants had health insurance.

#### Independent Variables

##### Financial difficulties

We measured financial difficulties using three items (α = 0.9) that asked how often participants lacked enough money for necessities, such as food, rent/mortgage, clothes, and utility bills. Responses for each item were given on a 5-point Likert- scale (1 never to 5 always) and a sum score was built ranging between 3 and 15, with higher scores reflecting more financial difficultie (lower SES).

#### Statistical Analysis

We used SPSS 22.0 (SPSS Inc., Chicago, IL, USA) to conduct the data analysis. Frequency (%) and mean (SD) were reported describing the sample. Bivariate correlations in the overall sample were calculated using Pearson's correlation. Linear regression models were applied with financial difficulties as the outcome, and the independent variables were gender, age, educational attainment, living alone, marital status, smoking, drinking, depressive symptoms, chronic pain, CMCs, SRH, and sick days. The list of variables was determined based on the literature review. Adjusted bivariate correlations controlled for gender, age, educational attainment, living alone, marital status, and health insurance as these variables fell under the category of demographic, social, and behavioral factors that had currently or in previous research demonstrated a relationship with financial difficulties. We controlled for these confounding variables in order to see the unique relationship between financial difficulties and mental and physical health outcomes. Smoking could not be included in the adjusted analysis as the linear regression included only continuous variables and smoking was measured as a dichotomous variable. We reported correlation coefficients (b), standard error (SE), 95% confidence intervals (95% CI), and *p*-values.

## Results

740 AA older adults who were at least 55 years old entered this study. Participants were on average 72 years old, with four CMCs. On average, they had considerable financial difficulty (9.18). They were mostly women (64%) and unmarried (86.5%). Most participants had never smoked and were not drinking alcohol (Table 1).

**Table 1:** Descriptive data.

**Characteristics**	**Mean**	**Std. deviation**
Age	71.73	8.37
Education	12.74	2.24
CMC (n)	4.23	2.12
SRH	3.13	1.02
Sick days	2.47	1.02
Depression	2.47	2.77
Pain	2.03	2.25
Binge frequency	0.49	1.38
Financial difficulty	9.18	5.64
	***n***	**%**
Gender		
Male	266	35.9
Female	474	64.1
Marital status		
Other	640	86.5
Married	100	13.5
Living alone		
No	294	39.7
Yes	446	60.3
Health insurance		
No	9	1.2
Yes	731	98.8
Smoking (ever)		
No	578	78.3
Yes	160	21.7
Drinking		
No	481	65.1
Yes	258	34.9
Binge drinking		
No	648	87.7
Yes	91	12.3

*CMCs, Chronic Medical Conditions; SRH, Self-Rated Health*.

### Unadjusted Correlates of Financial Difficulty

Table 2 shows the unadjusted (zero order) bivariate correlations between the study variables. Financial difficulty was inversely correlated with age, education, and marital status. Financial difficulty was positively correlated with CMCs (n), SRH (worse), sick days, depressive symptoms, chronic pain, smoking, drinking, any binge drinking, and number of binge drinking (Table 2).

**Table 2:** Unadjusted (zero order) bivariate correlations.

	**1**	**2**	**3**	**4**	**5**	**6**	**7**	**8**	**9**	**10**	**11**	**12**	**13**	**14**	**15**	**16**
1 Financial difficulty	1	−0.31^**^	−0.07	−0.08^*^	−0.07^*^	0.12^**^	−0.01	0.27^**^	0.26^**^	0.16^**^	0.43^**^	0.36^**^	0.24^**^	0.19^**^	0.12^**^	0.12^**^
2 Age		1	0.08^*^	−0.18^**^	−0.00	0.06	0.03	−0.07	−0.22^**^	−0.12^**^	−0.25^**^	−0.23^**^	−0.40^**^	−0.26^**^	−0.20^**^	−0.20^**^
3 Gender			1	0.11^**^	−0.12^**^	0.09^*^	−0.06	0.12^**^	0.01	0.01	−0.02	0.07	−0.19^**^	−0.11^**^	−0.20^**^	−0.20^**^
4 Education (years)				1	0.06	−0.04	0.04	−0.071	−0.03	−0.01	−0.07	−0.02	0.02	0.07	0.00	−0.02
5 Married					1	−0.41^**^	0.01	−0.049	−0.08^*^	−0.02	−0.07	−0.08^*^	−0.01	−0.06	0.03	0.03
6 Living alone						1	−0.02	0.10^**^	0.08^*^	0.04	0.12^**^	0.16^**^	0.01	0.07	0.02	0.01
7 Health insurance							1	0.064	−0.01	0.04	0.04	0.01	−0.04	−0.01	0.04	0.04
8 CMC (n)								1	0.30^**^	0.32^**^	0.41^**^	0.53^**^	0.03	0.05	0.00	0.02
9 SRH (1-5)									1	0.25^**^	0.37^**^	0.39^**^	0.18^**^	0.11^**^	0.10^**^	0.09^*^
10 Sick days										1	0.29^**^	0.34^**^	0.06	−0.01	0.05	0.04
11 Depressive symptoms											1	0.46^**^	0.21^**^	0.11^**^	0.08^*^	0.07
12 Pain (1–11)												1	0.14^**^	0.14^**^	0.08^*^	0.09^*^
13 Smoking (ever)													1	0.32^**^	0.34^**^	0.33^**^
14 Drinking (any)														1	0.50^**^	0.48^**^
15 Binge Drinking (any)															1	0.94^**^
16 Binge Drinking (n)																1

* p < 0.05;

** p < 0.01.

*CMCs, Chronic Medical Conditions; SRH, Self-Rated Health*.

### Adjusted Correlates of Financial Difficulty

Table 3 shows the adjusted (partial) bivariate correlations between financial difficulty, CMCs (n), SRH (1–5), sick days, binge drinking, and depressive symptoms. In these correlations, age, gender, education, marital status, insurance, and living alone were controlled. Financial difficulty was positively correlated with CMCs (n), SRH (worse), sick days, depressive symptoms, and chronic pain (Table 3).

**Table 3:** Adjusted (partial) bivariate correlations for continuous variables.

		**1**	**2**	**3**	**4**	**5**	**6**	**7**
1 Financial difficulty	r	1.00	0.21^**^	0.19^**^	0.12^**^	0.05	0.36^**^	0.29^**^
	p		0.000	0.000	0.002	0.218	0.000	0.000
	n	0	726	726	726	726	726	726
2 CMCs (n)	r		1.00	0.26^**^	0.27^**^	0.01	0.31^**^	0.45^**^
	p			0.000	0.000	0.754	0.000	0.000
	n		0	726	726	726	726	726
3 SRH (1–5)	r			1.00	0.23^**^	0.05	0.32^**^	0.35^**^
	p				0.000	0.192	0.000	0.000
	n			0	726	726	726	726
4 Sick days	r				1.00	0.01	0.26^**^	0.32^**^
	p					0.709	0.000	0.000
	n				0	726	726	726
5 Binge frequency	r					1.00	0.01	0.05
	p						0.779	0.163
	n					0	726	726
6 Depressive symptoms	r						1.00	0.41^**^
	p							0.000
	n						0	726
7 Chronic pain	r							1.00
	p							
	n							0

*p < 0.05;

** p < 0.01.

*CMCs, Chronic Medical Conditions; SRH, Self-Rated Health*.

## Discussion

The current study sought to understand mental and physical health correlates of financial difficulties in older AA adults living in economically disadvantaged areas of Los Angeles. The adjusted analysis, controlling for age, gender, education, marital status, living alone, and health insurance, showed that financial difficulties are correlated with depressive symptoms, chronic pain, number of CMCs, worse SRH, and sick days.

### Adjusted Correlates of Financial Difficulties

In the adjusted partial correlation analysis, we controlled for gender, age, education, marital status, living alone, and health insurance. Considering that health insurance is the gateway to healthcare and these demographic and SES factors are commonly correlated with health status (18, 43, 65–67), these variables were selected in order to analyze the unique relationship between financial difficulties and continuous mental and physical health variables. While much of the previous research would suggest that binge drinking is positively associated with financial difficulties, especially for AA samples (8, 9, 11), the findings did not indicate an association between financial difficulties and binge drinking. This may be due to the high percentage of participants who did not drink at all in our sample (65.1%). Considering the study environment is situated in economically disadvantaged areas, liquor store density is usually high and the influence of a substance use environment is evidenced to be stronger in the AA population (68). However, our convenience sample may have been skewed to include those who were more advantaged in the area rather than those who may have been more financially deprived or dealing specifically with substance use issues. Furthermore, those who binge drink more frequently are at an increased risk of earlier morbidity and therefore may not be included in our sample of older adults. Future research should explore whether this finding extends to other older AA adult samples and the possibility of age being a protective factor within this group.

Financial difficulties were also positively related to depressive symptoms. Much of the previous research has supported this relationship, showing a significant relationship between financial difficulties and the risk for depression as well as the severity of depressive symptoms (8, 14–16). Furthermore, research has found this relationship in AA samples and shown an increased risk among AAs and older adults (14–17). Considering the toll depressive symptoms take and the stress financial difficulties bring, it is understandable that these two variables are related, especially among a population that experiences disparities in income and mental health care (1, 69, 70). The current findings therefore align with the literature demonstrating a significant relationship between depressive symptoms and financial difficulties in older AA adults, and demonstrates that this relationship remains significant even after controlling for age, gender, education, marital status, living alone, and health insurance.

After controlling for the demographic and SES variables, financial difficulties were still positively associated with chronic pain in our sample. This aligns with much of the existing research which has shown that low SES is related to higher risk for incidence of pain, greater severity of pain, as well as, greater disability and distress from pain (18–22). While the previous research demonstrated the relationship between financial difficulties and several aspects of pain, the current study shows this relationship holds for AA samples specifically, who are especially at risk for pain mismanagement or under-treatment (71–74).

Additionally, after the adjusted analyses, financial difficulties were significantly positively associated with the number of CMCs. This finding aligns with past research indicating a link between financial difficulties and CMCs (23, 25). CMCs not only lead to increase medical costs but they detract from one's ability to work (25). Simultaneously, financial distress can take a toll on one's health physically, leading to an increased risk of CMCs (23). While we cannot make directional causal assumptions based on this analysis, these may be some of the underlying mechanisms behind the relationship between these two variables. These findings importantly demonstrate this association in an older AA sample, who have heightened rates of both financial difficulties and CMCs (1–4).

Furthermore, adjusted analyses revealed that financial difficulties were related to poorer SRH. This aligns with previous research revealing a relationship between financial difficulties or low-SES and SRH (26–29). While previous research has found this relationship in distinct low-income (28), AA (27), or older adult (29) samples, the current study provides evidence for this correlation among a sample with all of those characteristics. This provides more evidence for the relationship between financial difficulties and health variables like SRH for older AA adults, which is especially relevant given the limited health resources in low-income urban areas.

In addition, financial difficulties were positively associated with sick days. As suggested by limited previous research, financial difficulties and sickness absenteeism appear to be positively correlated in some populations (30–32). Given that taking sick days may lead to strains at work and thus financial difficulties, or that financial difficulties may take a toll on one's health leading to sick days, it is understandable that these two variables may be related although we cannot make directional causal assumptions based on the current analysis. Among this older population, managing multiple comorbidities may lead to several sick days when ill compared to younger populations who are usually healthier (75). Experiencing sick days can lead to multiple hospitalizations and visits, additional prescribed medications, and potential loss of income, if still employed (25). This can be overwhelmingly problematic for this AA population, especially if on a fixed income with costly health expenditures (76). Even if there is an acute period of a few sick days in an entire month, it can have a devastating effect on future financial outlooks for elderly individuals and their households (77).

These results are suggestive of a significant relationship between financial difficulties and various domains of mental and physical health (i.e., depression, chronic pain, CMCs, SRH, and sick days) of low-income AA older adults.

### Limitations

The findings of this study must be viewed within the context of its limitations. Due to the cross-sectional design, causal associations cannot be inferred. For both methodological and ethical reasons, it would be inaccurate to suggest financial difficulties are due to poor health or health behaviors in this population rather than entrenched societal inequalities. Although not studied here, financial difficulties in AA communities have historical and current links to institutionalized racism and systematic marginalization. Claims about the mechanisms underlying the associations identified here require more nuanced data and measurements collected over the life course to more adequately test the causal role of contextual and societal factors that may explain the observed associations.

Additionally, we did not have access to the clinical records of the participants to verify CMCs or mental health diagnosis nor did we have data on area level SES factors such as neighborhood income. We also did not have data on history of depression or other psychiatric disorders. Our results may be affected by self-report measurement bias. Future research should utilize administrative data or medical chart review when attempting to replicate these findings. Additionally, due to our non-random sampling, we cannot generalize our results to all AA communities. Despite these limitations, the current study furthers our understanding of mental and physical health correlates of financial difficulties among older AA adults in economically disadvantaged urban settings.

## Conclusions

The current study examined mental and physical health correlates of financial difficulties in a sample of older AA adults who were residing in low-income areas of South Los Angeles. After controlling for age, gender, education, marital status, living alone, and health insurance, financial difficulties were correlated with depressive symptoms, chronic pain, CMCs, SRH, and sick days. The findings shed light on the importance of screening for financial difficulties in addressing mental and physical health of older AA adults. Given that AA populations in similar settings (low-income areas) are facing economic disparities, the current study highlights that financial difficulties should be addressed in interventions designed to promote mental and physical health of AA older adults.

## Data Availability Statement

The datasets generated for this study are available on request to the corresponding author.

## Ethics Statement

The studies involving human participants were reviewed and approved by Institutional Review Board (IRB) Charles R. Drew University of Medicine and Science (CDU). The patients/participants provided their written informed consent to participate in this study.

## Author Contributions

ME prepared the first draft and revised the paper. MB designed the study, secured the funding, conducted the study, collected the data, and revised the manuscript. SA analyzed the data, conceptualized this report, and contributed to the revision of the paper. All authors contributed to the revision of the paper and approved the final draft.

### Conflict of Interest

The authors declare that the research was conducted in the absence of any commercial or financial relationships that could be construed as a potential conflict of interest.
